# ICD-11 and diabetes: opportunities for precision public health through enhanced classification

**DOI:** 10.3389/fpubh.2026.1944007

**Published:** 2026-10-01

**Authors:** Jingya Zhou, Peng Xia, Yuanmeng Li, Naishi Li

**Affiliations:** 1 Department of Medical Records, Peking Union Medical College Hospital, Chinese Academy of Medical Sciences and Peking Union Medical College, Beijing, China; 2 Collaborating Center for the WHO Family of International Classifications in China, Beijing, China; 3 State Key Laboratory of Complex Severe and Rare Diseases, Department of Nephrology, Peking Union Medical College Hospital, Chinese Academy of Medical Science and Peking Union Medical College, Beijing, China; 4 Key Laboratory of Endocrinology of National Health Commission of People's Republic of China, Department of Endocrinology, Peking Union Medical College Hospital, Chinese Academy of Medical Sciences and Peking Union Medical College, Beijing, China

**Keywords:** diabetes mellitus, disease classification, disease surveillance, health policy, ICD-11, postcoordination, precision public health, prediabetes

## Abstract

**Background:**

Diabetes mellitus poses a major global health challenge, yet its intrinsic heterogeneity has been poorly captured by traditional classification systems. The international classification of diseases, tenth revision (ICD-10), built on a phenotype-based framework, lacks the granularity needed to incorporate contemporary molecular insights into diabetes subtypes, hampering both clinical precision and public health surveillance.

**Main body:**

This review examines how the recently implemented international classification of diseases, eleventh revision (ICD-11) addresses these limitations through its ontology-based architecture. We analyze three key areas. First, ICD-11 reclassifies prediabetes as a distinct reportable entity with formal subtyping of impaired fasting glucose and impaired glucose tolerance, which may support more precise epidemiological tracking and evidence-based prevention. Second, its Uniform Resource Identifier (URI)-based taxonomy provides granular classification across the diabetes spectrum, from type 1 and type 2 diabetes to monogenic forms such as maturity-onset diabetes of the young (MODY), with etiological stratification that can inform both clinical decision-making and population-level surveillance. Third, its postcoordination mechanism allows multidimensional documentation of complications, drug-induced diabetes, and unstable disease states, generating structured, machine-readable data with potential for real-world evidence generation and health system performance assessment. However, early implementation experiences across multiple countries reveal substantial challenges—including heterogeneous adoption timelines, coding inconsistencies, and workforce training gaps—that may constrain the realization of these benefits in practice. We also discuss the implications of these advances for health systems, including value-based reimbursement models and the production of actionable evidence for policy makers.

**Conclusion:**

ICD-11 repositions diabetes classification toward a framework that offers substantially enhanced capacity for etiological and granular representation of the disease. By generating standardized, granular population-level data, it offers a potential infrastructure for precision public health. Yet realizing this potential will depend on health system capacity, workforce training, and sustained investment in implementation—particularly in resource-limited settings, where the risk of widening disparities is most acute. Future refinements should integrate genomic descriptors and dynamic update mechanisms to keep pace with rapid advances in diabetes research, with continued attention to health equity and global applicability.

## 1 Introduction

Diabetes has become one of the most pressing global public health problems. According to the international diabetes federation (IDF) diabetes atlas 11th edition, approximately 589 million adults (aged 20–79 years) are currently living with diabetes globally, and this number is projected to reach 853 million by 2050 (1). Recent IDF estimates indicate that 42.8% of individuals with diabetes, equivalent to 251.7 million adults, remain undiagnosed, placing them at higher risk of complications and delayed treatment (2). The disease and its complications are among the leading causes of death (3, 4), and annual healthcare expenditures have reached $1.3 trillion (5). The complexity of diabetes management arises largely from its heterogeneity. Traditional classifications fail to capture the full spectrum of the disease (6). For example, latent autoimmune diabetes in adults (LADA) is a slowly progressive condition that often masquerades as type 2 diabetes but carries autoimmune markers (7). Monogenic forms, such as maturity-onset diabetes of the young (MODY), stem from single-gene mutations affecting pancreatic β-cell function (8).

Standardized disease classification systems guide diagnosis and treatment while also ensuring reliable disease surveillance and data comparability for public health. The current International Classification of Diseases, Tenth Revision (ICD-10) system [first adopted in 1990 (9)], with updates until 2019 (10) categorizes diabetes into five major types within the E10–E14 block: type 1 (E10), type 2 (E11), malnutrition-related (E12), other specified (E13), and unspecified (E14). Gestational diabetes is classified separately under O24. But its phenotype-based classification, which relies largely on treatment response and clinical presentation, ignores contemporary molecular understanding of diabetes pathogenesis (11). MODY, for example, is grouped under “other specified diabetes” without gene-specific coding. This imprecision hinders targeted therapy, complicates risk assessment (12), and contributes to diagnostic inaccuracies worldwide.

ICD-11 aims to fix these problems with a new architecture (13). Its ontology-based design improves clinical accuracy and interoperability (14, 15), while its cluster coding mechanism captures multidimensional health states (14, 16). The impact goes beyond technical coding, however. ICD-10's coarse categories have real public health costs: they obscure the true distribution of diabetes subtypes, making targeted prevention hard to design and resources hard to allocate. The questions regarding ICD-11's implementation extend beyond coding accuracy: how will its improvements affect precise diabetes classification and personalized medicine; how can clinicians, public health professionals, and policymakers use ICD-11 across different settings, from clinical practice to epidemiological surveillance, population health management, and clinical trials; and what refinements are needed to maximize its public health utility? These considerations form the foundation for examining ICD-11's role in advancing diabetes care through a classification that bridges clinical utility with scientific progress and strengthens health systems' capacity to respond to this growing burden.

This review has three aims: to identify what ICD-10 gets wrong in diabetes classification; to assess how ICD-11's structure, particularly its etiological stratification and clinical subtyping, fixes those problems; and to consider how these changes could improve data collection and interoperability across health systems. We also propose recommendations for further refinements to ICD-11's diabetes classification framework and offer an evidence base to inform implementation strategies in diabetes-related clinical practice, public health surveillance, and health policy development, with attention to emerging scientific discoveries and evolving population health needs.

## 2 Methods

This narrative review synthesizes published literature and official classification documentation to examine the evolution of diabetes classification from ICD-10 to ICD-11 and its implications for precision public health.

### 2.1 Literature search strategy and evidence identification

A systematic literature search was conducted in PubMed, Web of Science (all databases and collections), and Scopus for publications from January 1, 2019, to December 31, 2025. The search strategy was constructed around two complementary thematic modules and applied across all databases, with syntax adapted as required by each platform. The first module targeted the intersection of ICD terminology and diabetes–related concepts, using the following free–text terms: “International Classification of Diseases,” “ICD-10,” “ICD-11,” “ICD-9,” “ICD9,” “ICD code,” and “diagnosis code,” combined with “Diabetes Mellitus,” “Prediabetic State,” diabet^*^, prediabet^*^, “type 1 diabetes,” “type 2 diabetes,” T1DM, T2DM, “diabetic complication,” “diabetes complication,” hyperglyc?emia, glycemic, and “metabolic syndrome.” The second module focused on the general ICD-11 literature, incorporating “ICD-11” and “International Classification of Diseases, 11th Revision.” The two modules were merged using the Boolean operator OR, with date restrictions limited to 2019–2025. The starting year of 2019 was selected to coincide with the formal adoption of ICD-11 by the World Health Assembly. The same conceptual framework was maintained across all databases, while the search syntax was tailored to each platform's specific requirements (e.g., field tags in PubMed, topic search in Web of Science, and TITLE–ABS–KEY in Scopus). Full search details are available from the corresponding author upon reasonable request. During the manuscript revision process, the literature search was supplemented to capture publications that appeared after the original cutoff date. Newly identified studies were assessed and, where relevant, incorporated into the discussion.

To complement the peer-reviewed literature with official technical and classification guidance, we systematically reviewed World Health Organization (WHO) documentation pertaining to ICD-11, including technical specifications, implementation frameworks, version release notes, and official reference materials. Specifically, we consulted the ICD-11 Reference Guide, the ICD-11 Implementation and Transition Guide, the complete series of “New in ICD-11” release updates spanning 2022 to 2025, and poster presentations from the WHO-Family of International Classifications (WHO-FIC) Annual Meetings held between 2020 and 2025. In addition, we screened the reference lists of retrieved reviews and guidelines to identify additional publications not indexed in the primary databases. All records identified from the database searches were merged and duplicates removed. Study selection was guided by relevance to the review's objectives, with priority given to studies reporting on: (1) ICD-11 coding practices and validation analyses; (2) diabetes-related classification applications; and (3) the practical challenges, health system preparedness, and policy frameworks associated with ICD-11 transition and implementation.

### 2.2 Classification version verification

All ICD-11 codes and entity names presented in this manuscript including those in the main text and Supplementary Table 1, were verified against the WHO ICD-11 for mortality and morbidity statistics (MMS) January 2025 frozen release (available at https://icd.who.int/browse/2025-01/mms/en). The January 2025 release was chosen as the reference because it represents the most recent version available at the time of manuscript drafting and analysis. Uniform Resource Identifiers (URIs) for entities presented in this manuscript were retrieved from the ICD-11 Foundation layer via the WHO-FIC Foundation maintenance platform (available at https://icd.who.int/dev11/f/en), as URIs are assigned to Foundation entities regardless of their codability in the MMS linearization. For ICD-10 references, we consulted the 2019 version (available at https://icd.who.int/browse10/2019/en).

## 3 Why ICD-10 falls short in diabetes classification

The international classification of diseases (ICD) is a standardized system for classifying diseases, injuries, and related health conditions, and is one of the core classifications within the WHO Family of International Classifications (WHO-FIC) (17). It is widely used for disease and mortality statistics, scientific research, healthcare management, and medical insurance reimbursement, and informs medical decision-making and resource allocation. ICD-10, released by the WHO in 1990 (9), has been the most widely adopted version worldwide for over three decades. The WHO updates the ICD approximately every 10 to 20 years to reflect advances in medical science and clinical practice (18). But the inherent limitations of ICD-10 are not fully remedied by its periodic updates (14). Integrating these changes is slow and complex, allowing many deficiencies to persist for extended periods. What is more, the outdated structure of ICD-10 cannot be updated through maintenance mechanisms to reflect contemporary advances in diabetes (19). Since ICD was originally designed for disease and mortality statistics, its classification granularity is coarse. This has created growing limitations in its application (20), such as the inability to accurately capture emerging clinical entities like monogenic diabetes and immune checkpoint inhibitor-associated diabetes (21), and to reflect many other subtypes of diabetes mellitus (DM). Over twenty countries have developed their own ICD-10 modifications (22–30). The variability makes cross-national comparisons difficult, especially for detailed diabetes complications and subtype data.

## 4 Key capabilities of ICD-11

ICD-11, formally adopted by the WHO in 2019, represents a fundamental shift from its predecessors (14, 15). Its digitally native, ontology-based architecture uses a uniform resource identifier (URI) system to assign a unique and persistent identifier to every clinical entity (15), thereby overcoming a critical limitation of ICD-10: the inability to support both broad statistical aggregation and detailed clinical subtyping at the same time. This framework works across multiple domains, including rare diseases (31), patient safety surveillance (32–34), and outcome-oriented healthcare, while integrating with modern electronic health environments.

ICD-11's advancements rest on three core components. First, its formal ontological architecture (the Foundation component) provides a rich semantic knowledge base that connects everyday clinical language with formal classification and ensures interoperability with systems like SNOMED CT (35). Second, a modular coding system using stem codes and extension codes can support flexible cluster codes (16, 36), allowing detailed representation of complex health conditions in both human-readable and machine-processable formats. Third, these features are embedded within a fully integrated digital ecosystem that includes an online Application Programming Interface (API), multilingual support, and advanced coding tools. The URI-based system ensures semantic consistency, facilitates analytics and real-world evidence generation, and maintains backward comparability. The ICD-11 for MMS, derived directly from this Foundation, is the official successor to ICD-10 for reporting purposes (14). Together, these capabilities make ICD-11 a foundational component for next-generation health information systems, which could support more accurate reimbursement, refined public health reporting, and enhanced capabilities for clinical care and biomedical research.

## 5 Prediabetes in ICD-11: from a lab finding to a reportable entity

Prediabetes, an intermediate hyperglycemic state between normal glucose regulation and diabetes, poses a major global public health challenge. According to the IDF Diabetes Atlas 11th Edition, approximately 635 million adults worldwide had impaired glucose tolerance (IGT) in 2024, and 488 million had impaired fasting glucose (IFG) (37). Annual progression rates to type 2 diabetes vary significantly across prediabetes subtypes, with individuals exhibiting both IFG and IGT facing a higher risk than those with isolated IFG (38, 39). This heterogeneity at the population level calls for a more precise classification system to facilitate early, subtype-specific interventions and to help health authorities monitor trends in these distinct high-risk populations.

Under ICD-10, prediabetes was categorized under “R73.0: abnormal glucose tolerance test” within Chapter XVIII (Symptoms, signs, and abnormal clinical and laboratory findings), so it was not recognized as a distinct pathophysiological entity (40). This limited recognition slowed clinical and public health responses (20), despite emerging evidence showing that prediabetes has measurable pathophysiological changes in multiple tissues and organs, making it a distinct pathogenic condition (41, 42). This was not just a semantic problem: it made prediabetes largely invisible in population-level disease registries and claims data, hindering systematic prevention strategies and equitable resource allocation.

The introduction of intermediate hyperglycemia (prediabetes) as an independent entity (Chapter 05) within ICD-11 represents a major nosological advancement. A key step is the formal incorporation of the clinically established distinction between Impaired Fasting Glucose (IFG, 5A40.0) and Impaired Glucose Tolerance (IGT, 5A40.1) into the global disease taxonomy (43). This differentiation is pathophysiologically grounded: IFG is primarily associated with hepatic insulin resistance (44), whereas IGT more strongly reflects peripheral (muscle) insulin resistance (45). Both IFG and IGT share pancreatic β-cell dysfunction. The rationale for this subcategorization extends beyond mechanistic insight; it provides a structured framework for risk stratification and shapes the evidence base for prevention (46–49). Landmark intervention trials, including the Diabetes Prevention Program (50), the Finnish Diabetes Prevention Study (51), and the Da Qing Diabetes Prevention Study (52), have robustly demonstrated that intensive lifestyle modification effectively reduces diabetes incidence in populations characterized predominantly by IGT. In contrast, the evidence from large-scale trials specifically targeting isolated IFG remains less conclusive, highlighting a need for further research to define optimal prevention strategies for this subgroup. This distinction has real implications for public health policy: it lets health systems direct proven lifestyle interventions toward the populations most likely to benefit, while also identifying evidence gaps that need future research.

By distinguishing IFG and IGT, ICD-11 may facilitate more precise epidemiological tracking of these heterogeneous high-risk states. This refined classification captures the distinct pathophysiology and varying strengths of preventive evidence associated with each subtype, which can in turn inform public health initiatives and resource allocation, ultimately supporting a more targeted and evidence-based approach to diabetes prevention at the population level.

## 6 Toward precision diabetes classification in ICD-11

Advances in genomics and molecular medicine have revealed the limitations of traditional diabetes classification, creating a demand for a more etiology-driven system. ICD-11 directly responds to this demand (12). However, prediabetes is only one part of the picture. For established diabetes, ICD-11 offers similarly significant advances in etiological classification. Traditional systems based on clinical features and insulin dependence are increasingly recognized as limited, failing to capture the disease's complex heterogeneity. The integration of multi-omics technologies (genomics, epigenetics, metabolomics) and AI-driven analytics has unveiled distinct molecular pathways underlying diabetes, which could support precise stratification and personalized management (12).

ICD-11 provides substantially greater capacity for etiological and granular representation of diabetes compared with its predecessor, though it does not constitute a fully molecular classification system. The ICD-11-MMS linearization categorizes diabetes into eight broad stem-code groups for statistical reporting: type 1 diabetes mellitus (T1DM, 5A10), type 2 diabetes mellitus (T2DM, 5A11), malnutrition-related DM (5A12), other specified diabetes mellitus (5A13), unspecified diabetes mellitus (5A14), diabetes mellitus in pregnancy (JA63), neonatal diabetes mellitus (KB60.2), and acute complications of diabetes (5A20–5A2Y). Under the category of “other specified diabetes mellitus,” the system further subdivides into nine subtypes based on underlying mechanisms. Beyond the MMS linearization, the ICD-11 Foundation layer encompasses over 60 distinct diabetes-related entities, each assigned a unique URI. Among these, approximately 30 are designated as reportable entities for statistical surveillance (i.e., those located above the “shoreline” in the MMS linearization); the remaining entities reside below the shoreline and, while retrievable via their URIs for detailed clinical documentation, are not independently codable in the MMS; instead, they are classified under a parent category that carries an MMS stem code in the Foundation hierarchy. This dual-layer architecture effectively balances the needs of epidemiological statistics with detailed clinical subtyping for optimal diabetes management. Due to its length, the complete table of diabetes entities, codes, and URIs is provided as Supplementary Table 1. The classification scheme for diabetes mellitus within the ICD-11 framework is shown in Figure 1.

**Figure 1:**
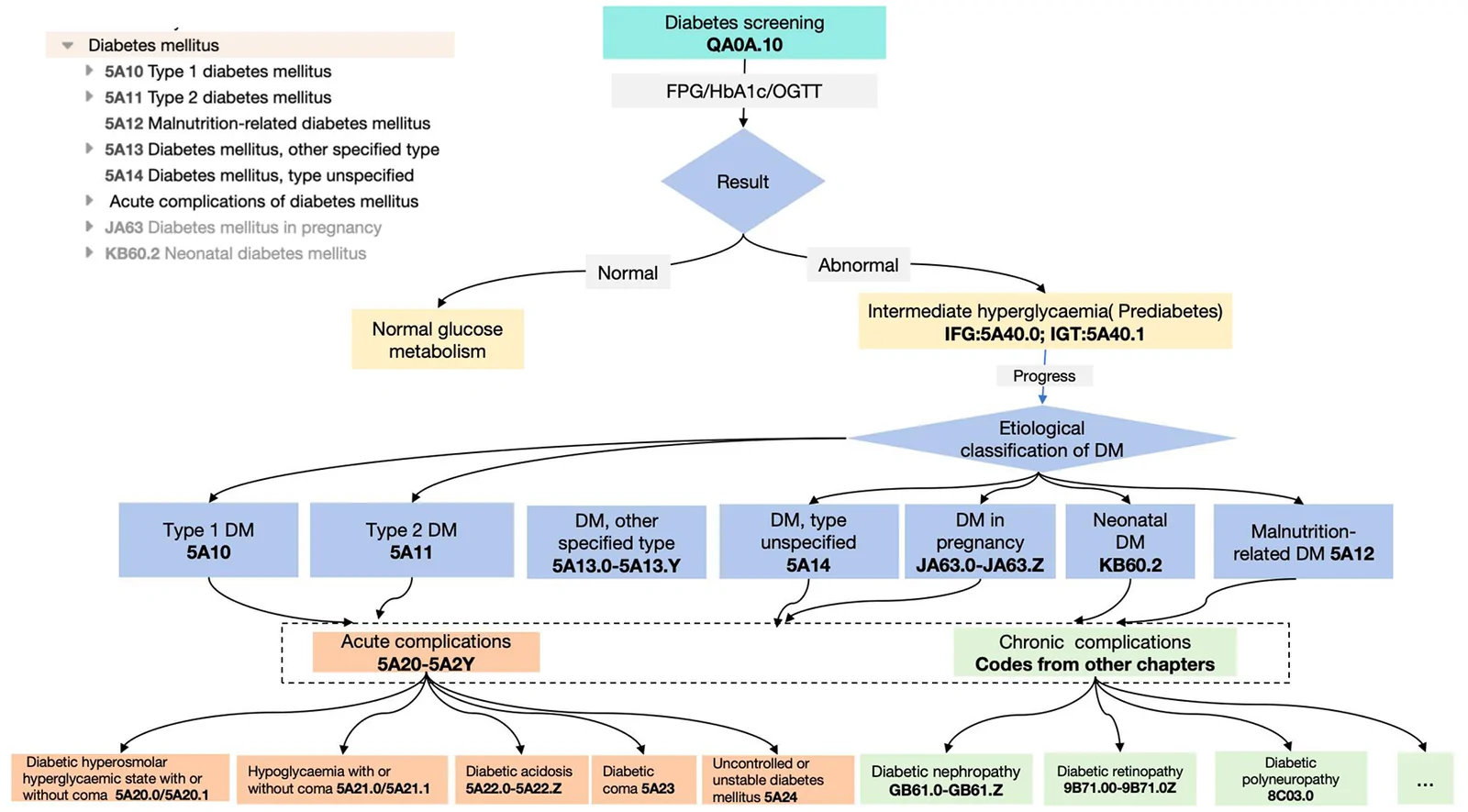
The classification scheme for diabetes mellitus in the ICD-11 framework. This diagram summarizes the ICD-11 diabetes classification framework, integrating the continuum from screening and diagnosis of intermediate hyperglycemia (prediabetes) to the major stem-code categories of diabetes mellitus and their associated acute and chronic complications. It demonstrates the framework's capacity to support a standardized, code-driven approach to the entire patient journey in diabetes care.

### 6.1 How URIs enable precision in diabetes classification

ICD-11 constitutes a significant evolution in the nosology of diabetes mellitus (DM). By introducing URIs for specific DM subtypes and related concepts, ICD-11 achieves a level of precision and machine-readability that could support its feasibility across varied healthcare contexts worldwide. This approach effectively mitigates a key limitation of prior classifications: the inadequate representation of the extensive clinical and pathophysiological heterogeneity within broad diabetic categories. The URI-based architecture creates a common semantic framework that could facilitate the aggregation of granular clinical data across institutions and national boundaries, offering a potential foundation for international collaborative research and comparative health system performance assessment.

All Foundation entities have a URI, which is a string of characters that uniquely identifies a particular ICD-11 entity, regardless of whether that entity is independently codable in the MMS linearization. In the ICD-11 architecture, the “shoreline” is a metaphor that distinguishes Foundation content from the entities extracted into derivative linearizations such as the MMS; importantly, the content and meaning of an entity are unaltered by this extraction (53). It is important to distinguish three tiers of granularity within the ICD-11 framework for diabetes, all of which derive from the same underlying ontology—the ICD-11 Foundation. First, entities that reside at or above the shoreline (e.g., 5A10 for T1DM, 5A11 for T2DM) are included in the MMS linearization and serve as the core reportable categories for official morbidity and mortality statistics. Second, Foundation entities that lie below the shoreline (e.g., the specific URI for LADA or for individual MODY subtypes) provide greater etiological precision but are not independently codable for statistical reporting; they can, however, be selected through the ICD-11 coding tool and, because all Foundation entities have URIs, they remain identifiable in structured data exports, ensuring they are not lost in aggregated statistics. Third, cluster coding through postcoordination allows users to combine multiple codes (36)—whether stem codes with extension codes, or multiple stem codes—to capture complex clinical details such as laterality, severity, complications, or causal relationships (e.g., linking a specific diabetes type to its complication or causative drug). Throughout the following subsections, we indicate which entities belong to each of these tiers to clarify what is reportable (stem codes), what is documentable (below–shoreline entities with URIs), and what is achievable through cluster coding, as summarized in Table 1. The availability of these tiers in routine administrative data varies by health system capacity and ICD-11 implementation status, as detailed in Table 1.

**Table 1:** ICD-11 granularity tiers for diabetes: entities and reportability.

Tier	Examples	Independently reportable in MMS?	Availability in routine administrative datasets
MMS stem codes	5A10 (T1DM), 5A11 (T2DM)	Yes	Directly available as diagnosis codes in routine administrative datasets
Below—shoreline Foundation entities	LADA (URI: http://id.who.int/icd/entity/1918279014)	No	Not available as standalone codes in routine administrative data; may be captured in structured data exports via URIs if coding tool is used, but not guaranteed in standard claims/reporting
Cluster coding through postcoordination	Diabetic coma due to type 1 diabetes mellitus(5A23/5A10);	Yes	Available only if health information system supports postcoordination; not universally available in all administrative datasets due to varying implementation capacity
	Type 1 diabetes mellitus with bilateral nonproliferative diabetic retinopathy(9B71.00&XK9J/5A11)		

#### 6.1.1 Type 1 diabetes mellitus

Although traditional diabetes classification systems offer broad categorical distinctions, significant heterogeneity within T1DM necessitates more granular subclassification. ICD-11 addresses this need through its Foundation layer, which enables detailed phenotypic characterization beyond the primary diagnostic code used for morbidity and mortality statistics. While ICD-11 for MMS provides a single entity for T1DM (code 5A10), the underlying ontological structure supports the specification of subtypes using URIs that reference unique clinical entities within the Foundation.

The system classifies T1DM into autoimmune (URI: http://id.who.int/icd/entity/1727622794) and idiopathic (URI: http://id.who.int/icd/entity/702044482) forms based on the presence or absence of pancreatic autoantibodies. ICD-11 also gives dedicated URIs to fulminant T1DM (URI: http://id.who.int/icd/entity/1237768242) and latent autoimmune diabetes in adults (LADA; URI: http://id.who.int/icd/entity/1918279014), reflecting differences in onset patterns and disease progression. The schema treats slowly progressive insulin-dependent diabetes mellitus (SPIDDM) as clinically synonymous with LADA, as both share the same URI. This refined subtyping framework aligns with contemporary understandings of T1DM heterogeneity and pathogenesis, supporting both clinical care and epidemiological research (54). The capacity to distinguish LADA from classic T1DM and T2DM through URI-based documentation may facilitate more detailed characterization of this subtype in clinical and research settings if such entities are systematically captured in structured data exports. As illustrated in Figures 2, 3, these subtypes are accessible within the MMS linearization as below-shoreline Foundation entities identifiable via their URIs, potentially enhancing the granularity and accuracy of diabetes classification in ICD-11.

**Figure 2:**
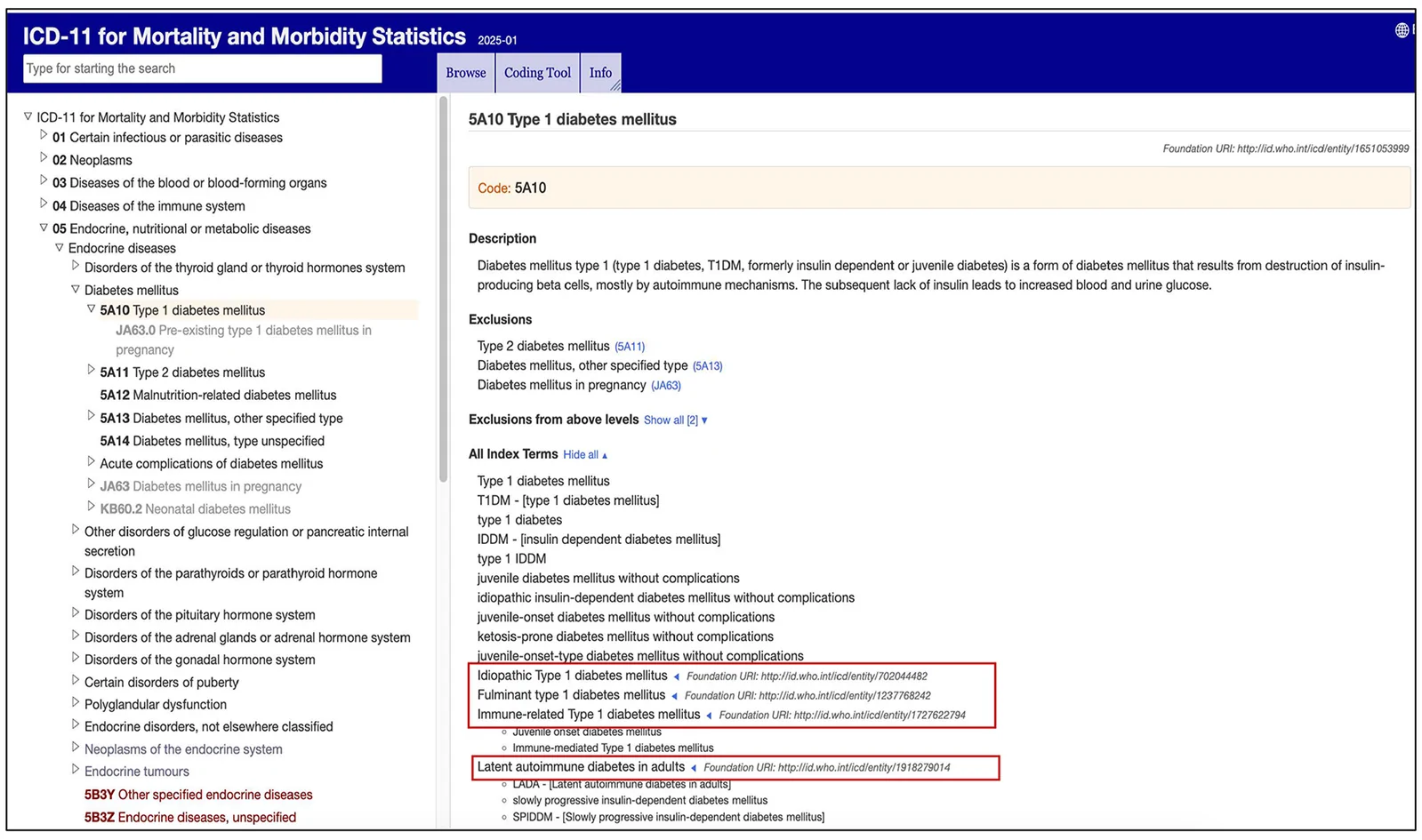
Specific subtypes of type 1 diabetes mellitus within the ICD-11 for MMS. These entities reside below the shoreline and are therefore not assigned individual classification codes. Instead, they are incorporated as index entries. When selected within the ICD-11 coding tool, these subtypes can be precisely retrieved and identified using their corresponding URIs. This enables granular data extraction while maintaining alignment with the official statistical reporting structure.

**Figure 3:**
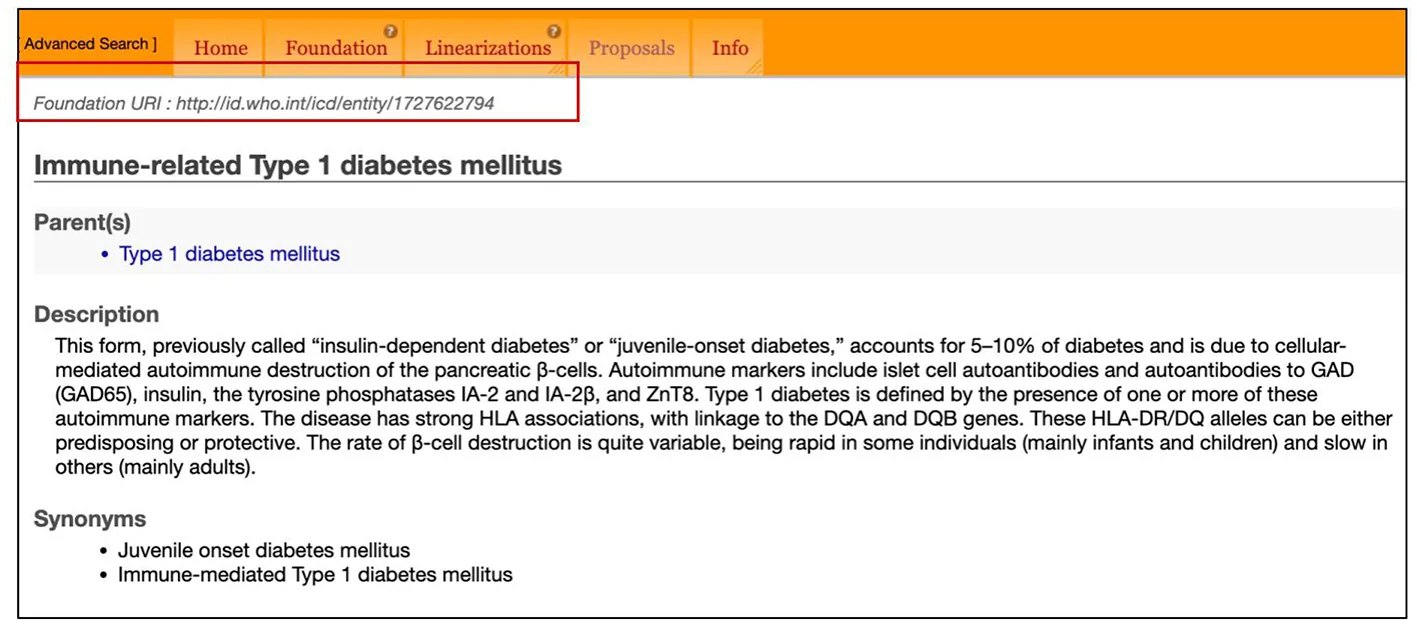
Foundation entity URI for the subtypes of diabetes mellitus taking immune-related type 1 diabetes mellitus as an example. This screenshot from the ICD-11 Foundation showcases the unique Uniform Resource Identifier (URI) for a clinical entity. The URI (highlighted) serves as a permanent, machine-readable web address that uniquely identifies “Immune-related type 1 diabetes mellitus” within the global ontology. It could support precise reference to this specific subtype, independent of language or coding linearizations, and may facilitate digital interoperability by linking clinical data, research, and terminology systems directly to the official WHO definition and its hierarchical position.

#### 6.1.2 Type 2 diabetes mellitus

The ICD-11 classification for T2DM retains 5A11 as the unified MMS stem code for epidemiological reporting. Within the Foundation layer, distinct clinical entities—such as “insulin-resistant diabetes” and “ketosis-resistant diabetes”— are assigned separate URIs and both map to the statistical code 5A11. These are Foundation entities rather than independently reportable MMS categories; neither can be used as a standalone code for official morbidity or mortality statistics, though both can be retrieved via the ICD-11 coding tool to support detailed clinical documentation when phenotypic subtyping is informative.

Importantly, the classification acknowledges phenotypic overlap across etiologies while maintaining distinct etiological categories. Diabetes due to confirmed genetic defects in insulin action (including insulin receptor mutations) is classified under 5A13.1 (“Diabetes mellitus due to genetic defects in insulin action”) within the “other specified diabetes mellitus” category, which is separate from and not subordinate to the T2DM (5A11) hierarchy. The URI-based Foundation layer enables precise documentation of these entities when the diagnosis is established, while the MMS parent code 5A11 serves as the reportable category for epidemiological aggregation.

Clinicians and coders should therefore distinguish between the reportable MMS stem code 5A11, the Foundation entities for detailed clinical documentation, and the distinct etiological category 5A13.1 for confirmed genetic insulin action defects. From a health equity standpoint, this pragmatic approach is particularly valuable in low- and middle-income countries, where advanced diagnostic capabilities are often scarce. By facilitating meaningful subtyping based on basic clinical examination, ICD-11 could help bridge the gap between evidence-based recommendations and real-world implementation, and thereby help mitigate the risk that patients in resource-limited settings are systematically disadvantaged in receiving subtype-informed care.

#### 6.1.3 Other specified diabetes mellitus: an etiology-driven framework

ICD-11 marks a deep restructuring in classifying “other specified diabetes mellitus,” moving from ICD-10's nonspecific single category to a detailed, etiology-based hierarchy. This new framework systematically categorizes distinct entities such as diabetes due to genetic defects of beta cell function (5A13.0), genetic defects in insulin action (5A13.1), diseases of the exocrine pancreas (5A13.2), endocrinopathies (5A13.3), drug- or chemical-induced diabetes (5A13.4), uncommon forms of immune-mediated diabetes (5A13.5), other genetic syndromes (5A13.6), clinically defined subtypes or syndromes (5A13.7), and other specified causes (5A13.Y).

This structure allows for granular categorization of monogenic diabetes, such as MODY subtypes 1–8, each with a dedicated URI, as well as pancreatic exocrine disorders, endocrinopathies, and syndromic forms such as Wolfram syndrome. This ontological structure, illustrated in Figure 4, can support genotype-directed management by providing a clear coding framework. For instance, patients with hepatocyte nuclear factor 1 alpha (HNF1A)-MODY (MODY 3) exhibit heightened sensitivity to sulfonylureas, making low-dose sulfonylureas the first-line therapy (8). Conversely, most patients with glucokinase (GCK)-MODY (MODY 2) have mild, stable hyperglycemia and rarely require pharmacological treatment (6). The coding system also captures rarer forms like thiamine-responsive megaloblastic anemia (TRMA) syndrome, where diabetes management involves high-dose thiamine supplementation (55). Beyond individual patient benefit, the granular classification of monogenic diabetes also has potential public health implications, particularly for cascade screening within families. For example, identifying a single case of MODY may prompt screening of relatives, potentially enabling earlier detection and intervention, which could help reduce the long-term burden of complications at the family and community level. This capacity illustrates how ICD-11 can contribute to precision public health by facilitating more targeted approaches to prevention and treatment.

**Figure 4:**
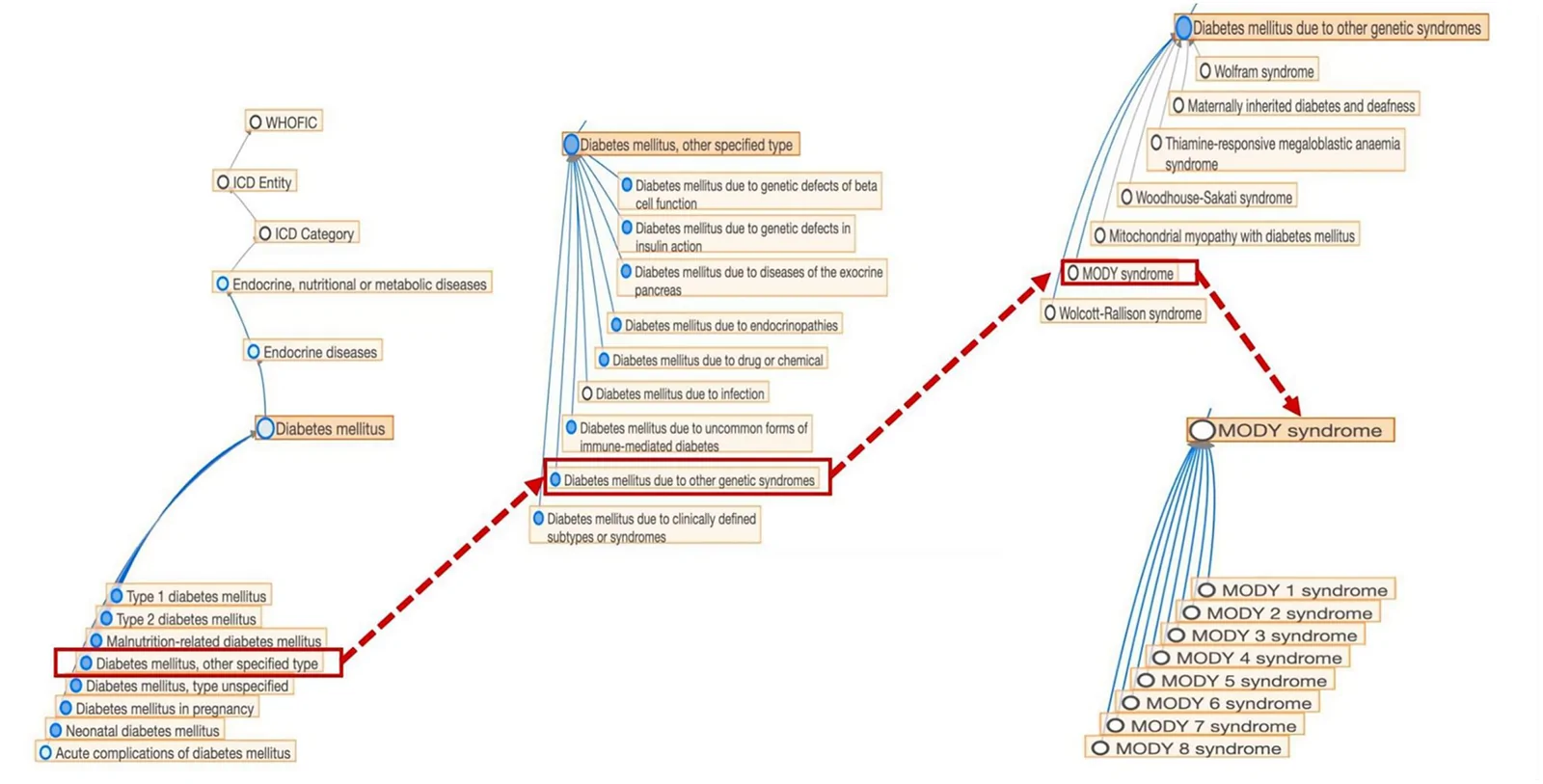
Hierarchical structure of ICD-11 Foundation for diabetes, exemplified by MODY syndrome. This hierarchical tree illustrates the ontological organization of diabetes mellitus and its subtypes within the ICD-11 Foundation layer. Blue circles represent codable entities designated for official mortality and morbidity statistics (MMS). White circles denote “below-shoreline” entities, which are non-codable for primary statistical reporting but are preserved in the ontology with unique URIs for precise clinical documentation and data retrieval. The structure demonstrates ICD-11's capacity for granular sub-classification, particularly within “other specified diabetes mellitus,” where distinct etiological subtypes, such as the various forms of MODY (e.g., MODY 1–8)— are systematically defined and logically linked, enabling a new level of specificity in diabetes phenotyping.

### 6.2 Better complication tracking through ICD-11 coding

ICD-11 represents a significant advancement in the management of diabetes complications through its sophisticated coding architecture, which allows for more precise documentation and may, in turn, support more proactive clinical decision-making. A key limitation of ICD-10 was its reliance on nonspecific categories, which grouped diverse acute and chronic complications under shared fourth-digit codes. In contrast, ICD-11 decouples diabetes types from their complications through a structured, flexible framework that better accommodates complex clinical scenarios and enhances big data analytics.

This new system utilizes postcoordination, allowing complications to be dynamically linked to specific diabetes subtypes via “has causing condition” linkages. This creates comprehensive patient profiles and enables accurate association of complications with distinct forms of diabetes, such as linking monogenic diabetes to its unique complication. Chronic complications are now systematically classified within their respective organ system chapters (e.g., diabetic retinopathy in ocular diseases, nephropathy in kidney diseases), promoting specialization in care. Acute complications, conversely, are consolidated under dedicated diabetes codes with enhanced specificity. For instance, standalone codes like 5A22.0 for diabetic ketoacidosis without coma could allow for precise prognostic evaluation and clear differentiation between distinct metabolic crises, such as diabetic ketoacidosis, hyperosmolar hyperglycemic state, and lactic acidosis, which were often conflated in ICD-10 (56).

The introduction of code 5A24, “Uncontrolled or unstable diabetes mellitus”—defined by WHO as describing “particularly hard-to-control Type 1 or Type 2 diabetes mellitus” that results in “frequent, extreme swings in blood glucose levels, causing hyperglycaemia that could lead to ketoacidosis or hypoglycaemia” (available at: https://icd.who.int/browse/2025-01/mms/en#1156295616)—addresses a critical documentation gap in ICD-10 where such high-risk states were invisible in claims data. The term “brittle diabetes” has historically been used to describe a condition of glycemic instability requiring frequent hospitalisations, impaired quality of life, and substantial economic cost (57). However, 5A24 should be regarded as a state of severe metabolic lability, not an acute metabolic emergency like diabetic ketoacidosis or severe hypoglycaemia. The code appears intended to identify patients with persistently difficult-to-control disease who require intensified chronic disease management, not those presenting with a single acute episode requiring emergency intervention. Its placement alongside acute complications in the ICD-11 structure may reflect the serious nature and high resource burden associated with this condition, without necessarily equating it with acute emergencies.

## 7 Postcoordination and real-world data generation for diabetes

ICD-11 provides a new framework for diabetes data standardization through its postcoordination mechanism, which enables precise, structured documentation of disease etiology, complications, and their causal linkages. Through semantic axes such as “has causing condition” and “has manifestation,” ICD-11 systematically links specific diabetes types (e.g., type 1, type 2) with their associated complications (e.g., retinopathy, neuropathy) in a structured, machine-readable format. This framework supports granular complication detailing by incorporating anatomical location, laterality, and clinical features (e.g., severity or the presence of gangrene), and thereby could enhance clinical specificity.

Another notable advancement is the precise coding of drug-induced diabetes using a “harm-cause-mode” model, which identifies the causative agent, the mechanism of injury, and the resulting adverse effect (32, 58). This allows clear documentation of complex scenarios, such as immune checkpoint inhibitor-induced diabetes (see Figure 5).

**Figure 5:**
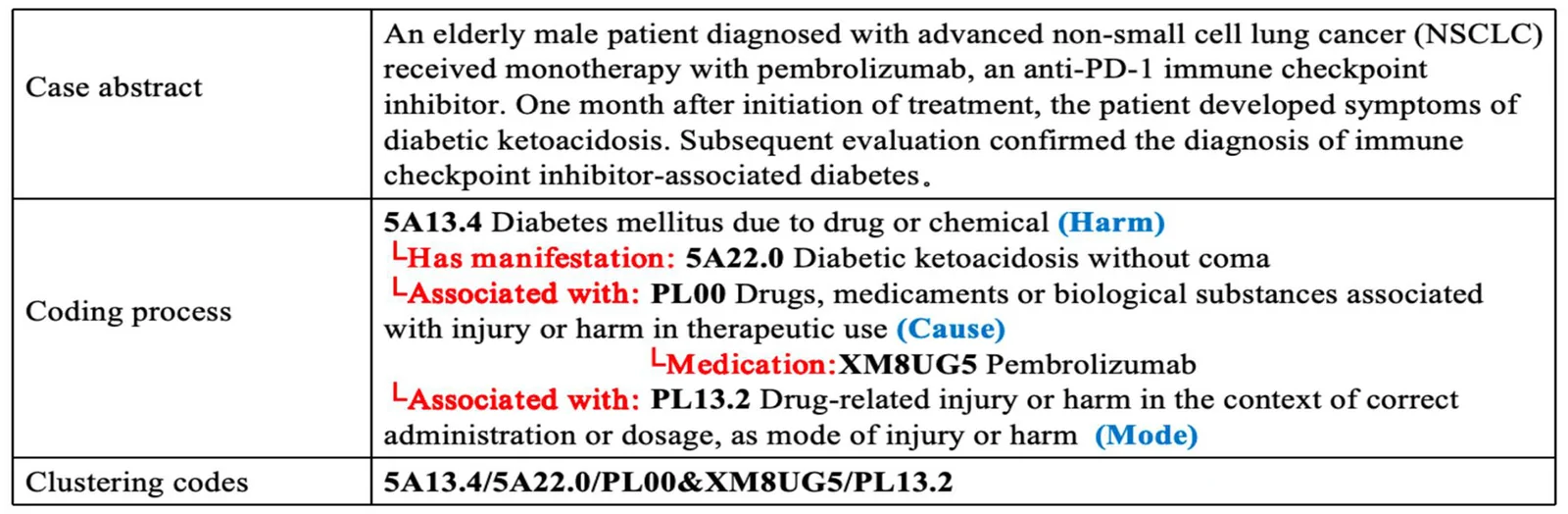
Coding example of immune checkpoint inhibitor-associated diabetes using ICD-11.

This multidimensional coding logic could facilitate high-resolution phenotyping of diabetes, which is crucial for large-scale data aggregation and real-world evidence generation. By transforming clinical documentation into structured, analyzable data, ICD-11 provides a foundation for data-driven approaches in diabetes care, potentially supporting advanced analytics, more personalized therapeutic strategies, and predictive modeling.

From a health systems perspective, this granular architecture creates new infrastructure for several important downstream applications. Theoretically, it could support automated risk stratification—for example, via electronic health record (EHR) alerts for codes such as “Uncontrolled or unstable diabetes mellitus” (5A24) to flag individuals requiring care intensification. It may also facilitate more accurate benchmarking of complication rates across institutions, and could inform the development of value-based reimbursement models tied to complication prevention. The structured data output is well-suited for generating real-world evidence to inform health policy, potentially supporting comparative effectiveness studies, population-level complication trend tracking, and evaluation of new therapies. As a globally standardized semantic framework, ICD-11 provides infrastructure for cross-border data sharing essential for international collaborative research and for monitoring global health trends in diabetes. However, it is important to recognize that these downstream applications remain potential rather than demonstrated outcomes of ICD-11 implementation. The translation of structured clinical data into improved population health outcomes ultimately depends on health system capacity, data governance frameworks, and integration with clinical workflows—factors that extend beyond the classification system itself.

Emerging proof-of-concept studies illustrate the feasibility of translating structured health data into population-level risk stratification. For instance, Pavlou et al. developed an informatics-based, data-led prioritization strategy using routinely collected EHR data to enable objective and equitable risk stratification in an ethnically diverse urban cohort with type 1 diabetes (59).While their approach did not directly use ICD-11 codes, it illustrates the type of data-led risk stratification that ICD-11's granular architecture is intended to support. However, empirical evidence directly linking ICD-11 implementation to such outcomes is still needed. This ontological framework is visually summarized in Figure 6 (ICD-11 Diabetes Coding Logic Atlas), which illustrates the dynamic integration of etiology, complications, and drug-related effects.

**Figure 6:**
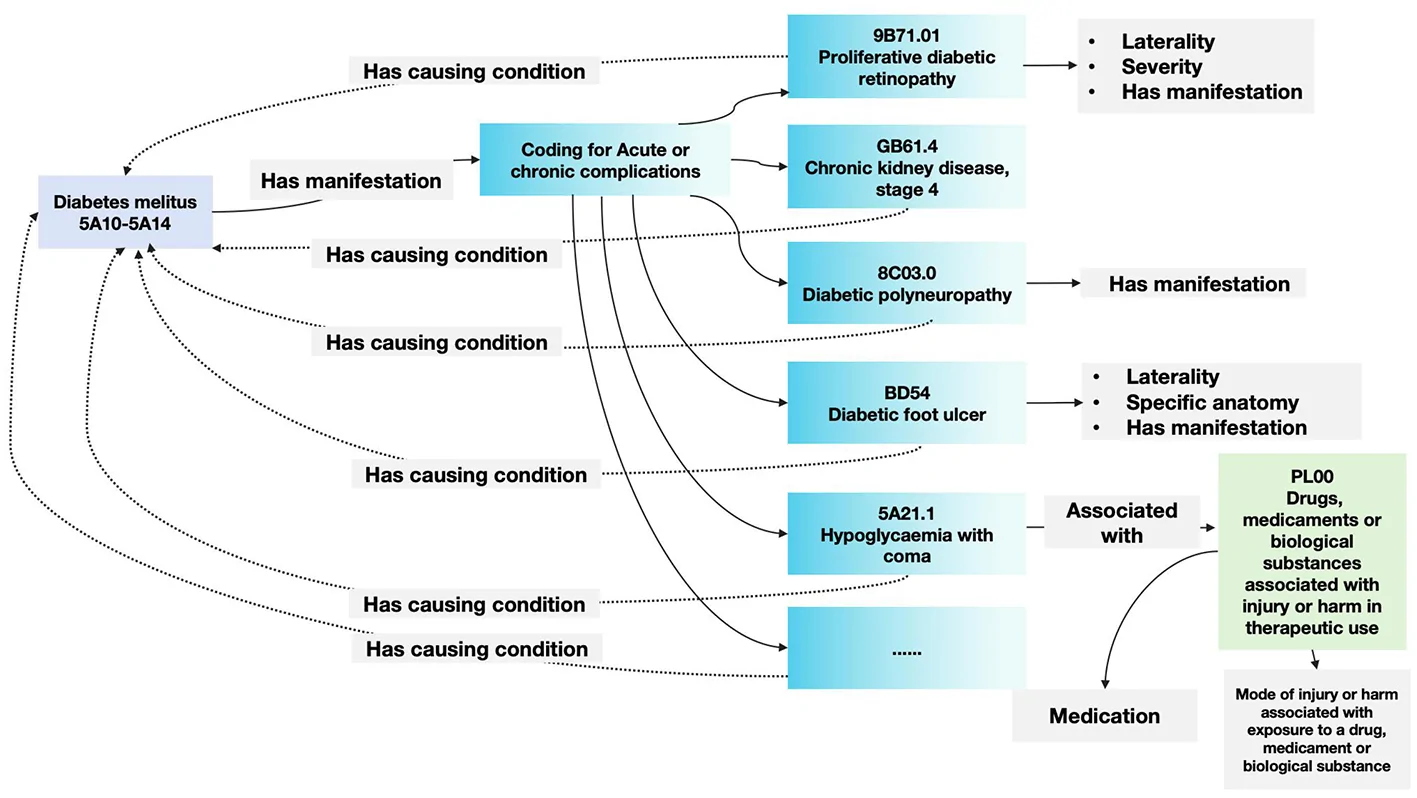
ICD-11 Diabetes Coding Logic Atlas using postcoordination for multidimensional coding. This diagram visually demonstrates how ICD-11's ontological structure enhances diabetes classification through multi-axis relationships and postcoordination capabilities. Light gray boxes indicate postcoordination axes (e.g., etiology, severity, and drug associations), which allow for dynamic combination with stem codes to capture clinical complexity.

## 8 Remaining challenges and opportunities for ICD-11

Widespread adoption of ICD-11 faces real-world challenges that extend beyond cost, training, and user resistance. These challenges span technical, organizational, and health equity dimensions, and their interplay will ultimately determine whether ICD-11 fulfills its potential as a tool for precision public health.

### 8.1 Heterogeneous adoption and transitional arrangements

ICD-11 was released in May 2019 and recommended for global use from January 2022 (14), yet implementation remains highly uneven across countries. According to WHO data from May 2024, the global implementation of ICD-11 is steadily advancing, with 132 Member States and areas at various phases of adoption—specifically, 72 countries have begun implementation, and 50 countries are conducting or expanding implementation pilots (9). National roadmaps illustrate this diversity: Australia targets 2031 for ICD-11 as the standard in health data reporting, with an in-principle decision expected by late 2026 (60, 61); Korea plans pilot implementation in 2028 and full adoption by 2031, following a 2025 field trial that revealed significant coding challenges (62); Brazil aims for nationwide implementation by 2027 through a phased five-axis strategy (63); Japan targets 2027 for mortality coding, while addressing workforce capacity, translation, and extension code standardization (64); and Mexico, one of the early adopters in the Americas, is integrating ICD-11 into national health information systems including the birth information subsystem (65, 66). This heterogeneity complicates cross-national comparisons, as some jurisdictions will continue to rely on ICD-10 or national modifications—such as ICD-10-AM (Australia), ICD-10-GM (Germany), ICD-10-CA (Canada), and ICD-10-KM (Korea) (22–27)—for extended periods. The transition itself requires systematic mapping from ICD-10 and national modifications to ICD-11, a process that is technically demanding and imperfect. Fung et al. examined the feasibility of replacing ICD-10-CM with ICD-11 for U.S. morbidity coding, demonstrating that a stepwise strategy using stem codes, Foundation entities, and postcoordination could achieve full representation of nearly 90% of codes, though 5.5% of codes were not one-to-one matches, posing practical transition challenges (67). A multi-country automated mapping study using CodeFusion compared six national ICD-10 modifications (US, Canada, Australia, France, China, and Sweden) and revealed that match rates to ICD-11 varied substantially: Canada and Australia achieved 84% and 79% respectively, while ICD-10-CM (US) had a lower match rate of 56%, albeit with more postcoordination matches (68). In a Canadian study applying WHO mapping tables to mortality data, 96.6% of ICD-10 codes for adults could be mapped to ICD-11, yet gaps remained for certain conditions (69). In cancer registration, mapping from ICD-10 to ICD-11 has revealed inconsistencies, particularly for complex diagnoses requiring postcoordination (70). During the transition, many health systems will need to operate dual coding (capturing both ICD-10 and ICD-11 codes simultaneously) to maintain longitudinal data comparability, imposing additional workload and system costs (71). A Korean field trial of dual coding with 172 cases and 815 coding lines found that overall ICD-11 code agreement was only 34.2%, with agreement dropping to 22.9% for codes requiring postcoordination (62). Malaysia has explored automated coding approaches to facilitate this transition, though challenges remain in integrating these tools with existing health information infrastructures (72).

### 8.2 Technical integration, data quality, and governance

Effective ICD-11 implementation demands robust integration with existing EHR systems and APIs. A pilot implementation in West China found that while coders acknowledged ICD-1's improvements, the coding process was time-consuming, with 41.10% of codes requiring postcoordination and some cases needing up to eight codes (73). Critically, the quality of medical record documentation frequently did not meet ICD-11 requirements—underscoring that ICD-11's granularity may be constrained by the underlying clinical data quality (73, 74). In Bangladesh, a pilot integrating the ICD-11 API into an Open Medical Record System (OpenMRS)-based hospital automation system was successfully deployed across three district hospitals; however, the pilot revealed several challenges, including physician sequencing errors, nurse data-entry inconsistencies, and functional gaps in the inpatient module under development (75). A feasibility study in Malaysia using the consolidated framework for implementation research (CFIR) identified additional challenges: outdated infrastructure, potential for data duplication and limited data validity, inadequate workforce capacity, steep learning curves for vendors, and insufficient funding for system-wide transitions (76). These findings underscore that governance and information-system requirements—including clear roles, data stewardship frameworks, and quality assurance mechanisms—must be addressed before ICD-11 can deliver reliable population-level data.

### 8.3 Coder and clinician training

The learning curve for mastering postcoordination and URI-based coding is steep—a challenge that becomes evident when examining implementation experiences across different countries. Training needs extend beyond professional coders to clinicians, whose documentation practices directly determine coding quality. A field trial in Korea identified that coder experience and training levels significantly affected coding consistency, with discrepancies arising between ICD-10 and ICD-11 coding approaches and among coders (74). A Malaysian study evaluating ICD-11 training effectiveness further confirmed that while training improved coder knowledge and confidence, sustained competency required ongoing support beyond initial instruction (77). International experience provides further insights: in Japan, the Japan Society of Health Information Management has conducted 13 in-person and 4 online training sessions since 2019, reaching 7,308 participants, with approximately 55% of attendees being first-time learners of ICD-11 (64). In Iran, a nationwide three-phase blended training program (online modules, offline materials, and in-person Training of Trainers sessions) for approximately 600 mortality coders across 31 provinces found that virtual training alone was insufficient for developing practical coding skills, and that face-to-face instruction remained essential (78, 79). Lessons from Argentina and Mexico—where ICD-11 preparatory work has focused primarily on mortality coding—emphasize the need for concise manuals, instructor guides, and tailored programs, alongside a shortage of specialists to deliver training (13). The timely release of guidelines and training materials in multiple languages is critical; delays in the release of Spanish-language versions, for example, have been reported as a barrier to implementation in some settings (80). France has taken an innovative approach by designing training programs specifically for software vendors, using stakeholder consultations and iterative curriculum development to address the varying levels of expertise among vendors (81).

### 8.4 Completeness and reproducibility of postcoordination

ICD-11's postcoordination mechanism offers unprecedented flexibility, but it also introduces risks of inconsistent application across coders and institutions. The cancer registry study noted the lack of validation mechanisms that allow invalid combinations of stage, grade, and morphology codes, potentially compromising data quality (70). Zarei et al. (82) compared the accuracy of inpatient morbidity coding between ICD-11 and ICD-10, reporting that coding accuracy varied by condition and highlighting the need for robust training and validation protocols to ensure reproducible postcoordination. A Korean field trial found that discrepancies in coded results were more likely for diagnoses requiring multiple codes or postcoordination (74), while the West China pilot reported that some cases needed up to eight codes to fully capture clinical detail (73). In Chile's 2025 national pilot of ICD-11 morbidity coding across 457 hospital discharges, the gold standard identified 33 errors in main diagnoses, of which 97% were omissions of postcoordination—highlighting that even experienced coders require focused training on postcoordination (83). The UK Office for National Statistics, during its preparatory work for mortality coding, similarly found variation in extension code application among coders, noting that extension codes are not mutually exclusive and the same concept can be reflected with multiple codes (84). Establishing standardized protocols for postcoordination use, accompanied by automated validation tools, will be essential to ensure that the granularity gained through ICD-11 translates into reproducible, comparable data rather than increased variability.

### 8.5 Backward compatibility and longitudinal datasets

A major concern for health systems and researchers is backward compatibility with ICD-10-coded historical data. Longitudinal datasets spanning decades—particularly mortality registries and chronic disease cohorts—must remain interpretable across the ICD-10/ICD-11 transition. WHO provides mapping tables, but as the Alberta study demonstrated, mapping coverage is not universal (69). A study using Shannon entropy to evaluate information changes during transition highlighted that the structural differences between ICD-10 (tree-type hierarchical) and ICD-11 (polyhierarchical ontology) make direct comparisons challenging (85). Canada's assessment of over 100 national health indicators found a high rate of equivalency from ICD-10-CA to ICD-11, but identified cases where postcoordination would be required to maintain specificity, as well as conditions that could only be mapped to Foundation-level URIs rather than reportable MMS codes (86). Health systems will need to invest in developing and validating mapping algorithms for their specific data contexts, including the use of transitional or dual coding during a period of parallel operation (71).

### 8.6 Health equity and the risk of widening disparities

Arguably the most profound concern is whether ICD-11's increased coding granularity will widen rather than reduce inequalities between well-resourced and resource-limited healthcare systems. High-income settings with mature EHR infrastructure, trained coding specialists, and access to genetic testing can fully leverage ICD-11's etiological detail. In contrast, low- and middle-income countries may struggle with outdated infrastructure, limited workforce, and insufficient funding for system-wide transitions (76). Yet innovative approaches are emerging. Zimbabwe's integration of ICD-11 into the District Health Information Software 2 (DHIS2) platform demonstrates a path forward for resource-limited settings, embedding ICD-11 coding directly in data entry forms to strengthen data quality and reduce manual reporting (87). Bangladesh's pilot of ICD-11 API integration with OpenMRS shows how open-source platforms can lower implementation barriers, though challenges of internet connectivity and limited training capacity remain (75). Iran, despite economic sanctions and funding constraints, successfully trained 600 mortality coders across 31 provinces using a cost-effective Training of Trainers model that combined online and in-person instruction (78). Rwanda has deployed a Docker-based localized ICD-11 instance to reduce dependency on internet connectivity, enabling automated underlying cause of death selection through WHO digital open rule integrated cause of death selection (DORIS) algorithms even during internet disruptions (88). The UNRWA experience further illustrates that successful implementation in humanitarian settings depends on external policy support, leadership commitment, and effective change management (89). Taken together, these examples suggest that resource-limited settings can overcome barriers through local innovation and international support—yet without deliberate investment in capacity-building, the promise of precision public health may inadvertently be realized only for those populations already best served, a risk that demands explicit attention from WHO and global health partners. The experience of Brazil, for instance, illustrates how regional leadership can help bridge this gap, as the only Portuguese-speaking WHO Collaborating Center leading translation and capacity-building efforts for the Community of Portuguese Language Countries (63).

Acknowledging these challenges is essential for realistic implementation planning. Yet it is equally important to recognize that ICD-11 is not a static classification—its underlying infrastructure already includes mechanisms designed to support ongoing evolution. Despite these implementation barriers, the current ICD-11 framework is already equipped with an established maintenance and update mechanism, including the online Proposal Platform through which registered users can submit evidence-based proposals for adding new entities, refining content models, or adjusting hierarchical relationships. Evidence of this platform's activity is substantial: from July 2014 to July 2024, a total of 20,600 proposals were submitted, of which approximately 14,500 (70.4%) were implemented or implemented with modification (90). Given that ICD-11 already has this infrastructure in place, the more relevant question is not whether a new update mechanism should be created, but rather how rapidly and systematically emerging genomic entities can be incorporated into the existing process. As emphasized by Redondo and Balasubramanyam (91), improved etiological classification is essential to advance “precision diabetes.” To this end, several measures could accelerate the incorporation of new genomic knowledge into ICD-11.

First, for monogenic diabetes, adopting gene-specific nomenclature (e.g., GCK-monogenic diabetes, HNF1A-monogenic diabetes) in place of phenotype-based terms such as MODY would better reflect underlying molecular pathology and support genotype-directed management (19). Second, to bridge clinical coding with genomic medicine, ICD-11 should harmonize with standardized genomic ontologies and databases, such as the nomenclature system of the Human Genome Variation Society (HGVS) (92) and the clinical variant database ClinVar (93). Third, active engagement of specialty societies in submitting proposals for newly characterized monogenic diabetes subtypes—alongside systematic literature reviews by WHO-FIC collaborating centers to identify evidence gaps—could enhance the efficiency and responsiveness of the maintenance workflow, from proposal submission to review by the Classification and Statistics Advisory Committee (CSAC) and Medical and Scientific Advisory Committee (MSAC), to final implementation in the annual frozen release. Finally, for diabetic complications, incorporating detailed qualifiers such as Wagner grade severity stages for diabetic foot syndrome within the postcoordination framework would enable more precise phenotyping of complication severity.

## 9 Conclusion

Returning to the three objectives outlined in the introduction, this review has demonstrated that: (1) ICD-10's framework is indeed inadequate for capturing the contemporary understanding of diabetes heterogeneity; (2) ICD-11 addresses these limitations through its etiology-driven architecture, URI-based granularity, and postcoordination mechanisms; and (3) these advancements create new possibilities for standardized data collection, interoperability across healthcare systems, and real-world evidence generation.

The transition from ICD-10 to ICD-11 marks a paradigm shift in diabetes classification, moving from a predominantly phenotype-based framework toward one that offers substantially enhanced capacity for etiological and granular representation of the disease, better capturing its molecular and phenotypic heterogeneity while maintaining the practical constraints of a global statistical classification system.

By more accurately reflecting the complexity of individual diabetes cases, ICD-11 could support individualized treatment selection, potentially improve prognostic stratification, and could facilitate refined research cohort design, and may help develop more equitable reimbursement models, including Diagnosis-Related Group (DRG) payment system. But its value extends beyond clinical precision to population health. By generating accurate, comparable, and actionable population-level data, ICD-11 could support precision public health by facilitating the tailoring of prevention strategies to specific subpopulations based on their risk profile; it could support equitable health systems, ensuring that the classification framework serves the needs of diverse global populations from high-resource settings to low- and middle-income countries; and it could provide the data infrastructure needed for evidence-based policy, including value-based reimbursement, quality benchmarking, and health resource allocation.

Combining structured postcoordination with URIs, ICD-11 can facilitate interoperability with EHRs and external clinical terminologies. The system can function at different levels of diagnostic sophistication. In settings where advanced testing is unavailable, the system remains usable, allowing accurate classification based on robust clinical phenotypes and supporting global applicability. By bridging evolving scientific knowledge with routine clinical practice, ICD-11 offers a basis for personalized diabetes care and provides a framework for future innovations in genomic medicine and population health management.
