# Supplementary material for: ICD-11 and diabetes: opportunities for precision public health through enhanced classification
Source: Front Public Health. 2026 Oct 1;14:1944007. doi: 10.3389/fpubh.2026.1944007 (PMC13670241; doi:10.3389/fpubh.2026.1944007)
Supplement: Supplementary file 1 [file Supplementary_file_1.docx]

| **Supplementary Table 1. Diabetes mellitus entities: nomenclature, codes and URIs in ICD-11** | | | |
| --- | --- | --- | --- |
| **Level** | **Entity name** | **Code** | **URI** |
| **Category** | **Type 1 diabetes mellitus** | **5A10** | **1651053999** |
| Foundation entities | Idiopathic Type 1 diabetes mellitus | 5A10 | 702044482 |
|  | Fulminant type 1 diabetes mellitus | 5A10 | 1237768242 |
|  | Immune-related Type 1 diabetes mellitus | 5A10 | 1727622794 |
|  | Latent autoimmune diabetes in adults | 5A10 | 1918279014 |
| **Category** | **Type 2 diabetes mellitus** | **5A11** | **119724091** |
| Foundation entities | Ketosis-resistant diabetes mellitus | 5A11 | 618467158 |
|  | Insulin resistant diabetes mellitus | 5A11 | 1255683285 |
| **Category** | **Malnutrition-related diabetes mellitus** | **5A12** | **1150913605** |
| **Category** | **Diabetes mellitus, other specified type** | **5A13** | **381961554** |
| **Subcategory** | **Diabetes mellitus due to genetic defects of beta cell function** | **5A13.0** | **1484144649** |
| **Subcategory** | **Diabetes mellitus due to genetic defects in insulin action** | **5A13.1** | **133436462** |
| **Subcategory** | **Diabetes mellitus due to diseases of the exocrine pancreas** | **5A13.2** | **1050647875** |
| **Subcategory** | **Diabetes mellitus due to endocrinopathies** | **5A13.3** | **1736581746** |
| **Subcategory** | **Diabetes mellitus due to drug or chemical** | **5A13.4** | **388963453** |
| Foundation entities | Steroid-induced diabetes | 5A13.4 | 235906657 |
| **Subcategory** | **Diabetes mellitus due to uncommon forms of immune-mediated diabetes** | **5A13.5** | **1418416880** |
| **Subcategory** | **Diabetes mellitus due to other genetic syndromes** | **5A13.6** | **1730828870** |
| Foundation entities | MODY syndrome | 5A13.6 | 419255568 |
|  | MODY 1 syndrome | 5A13.6 | 289825291 |
|  | MODY 2 syndrome | 5A13.6 | 990655222 |
|  | MODY 3 syndrome | 5A13.6 | 964882179 |
|  | MODY 4 syndrome | 5A13.6 | 679107840 |
|  | MODY 5 syndrome | 5A13.6 | 566324219 |
|  | MODY 6 syndrome | 5A13.6 | 2096037122 |
|  | MODY 7 syndrome | 5A13.6 | 1745614099 |
|  | MODY 8 syndrome | 5A13.6 | 205210166 |
|  | Wolcott-Rallison syndrome | 5A13.6 | 2096915129 |
|  | **Diabetes mellitus due to clinically defined subtypes or syndromes** | **5A13.7** | **891371477** |
| **Subcategory** | **Diabetes mellitus due to other specified cause** | **5A13.Y** | **/** |
| Foundation entities | Diabetes mellitus due to infection | 5A13.Y | 1823568169 |
| **Category** | **Diabetes mellitus, type unspecified** | **5A14** | **/** |
| **Category** | **Acute complications of diabetes mellitus** | **/** | **1304552818** |
| **Subcategory** | **Diabetic hyperosmolar hyperglycaemic state** | **5A20** | **463969617** |
| **Subclassification** | **Hyperosmolar hyperglycaemic state without coma** | **5A20.0** | **1322661241** |
| **Subclassification** | **Hyperosmolar hyperglycaemic state with coma** | **5A20.1** | **1796958395** |
| **Subclassification** | **Diabetic hyperosmolar hyperglycaemic state, unspecified** | **5A20.Z** | **/** |
| **Subclassification** | **Hypoglycaemia in the context of diabetes mellitus** | **5A21** | **193526903** |
| **Subclassification** | **Hypoglycaemia in the context of diabetes mellitus without coma** | **5A21.0** | **1153329797** |
| **Subclassification** | **Hypoglycaemia in the context of diabetes mellitus with coma** | **5A21.1** | **1973651647** |
| **Subclassification** | **Hypoglycaemia in the context of diabetes, unspecified** | **5A21.Z** | **/** |
| **Category** | **Diabetic acidosis** | **5A22** | **2144077558** |
| **Subcategory** | **Diabetic ketoacidosis without coma** | **5A22.0** | **540530695** |
| **Subcategory** | **Diabetic lactic acidosis** | **5A22.1** | **58193584** |
| **Subcategory** | **Diabetic metabolic acidosis** | **5A22.2** | **493397722** |
| **Subcategory** | **Diabetic ketoacidosis with coma** | **5A22.3** | **939674472** |
| **Subcategory** | **Other specified diabetic acidosis** | **5A22.Y** | **/** |
| **Subcategory** | **Diabetic acidosis, unspecified** | **5A22.Z** | **/** |
| **Category** | **Diabetic coma** | **5A23** | **367815473** |
| **Category** | **Uncontrolled or unstable diabetes mellitus** | **5A24** | **1156295616** |
| Foundation entities | Brittle diabetes mellitus | 5A24 | 23809728 |
| **Category** | **Other specified acute complications of diabetes mellitus** | **5A2Y** | **/** |
| **Category** | **Diabetes mellitus in pregnancy** | **JA63** | **169360210** |
| **Subcategory** | **Pre-existing type 1 diabetes mellitus in pregnancy** | **JA63.0** | **1240893990** |
| **Subcategory** | **Pre-existing type 2 diabetes mellitus in pregnancy** | **JA63.1** | **750602160** |
| **Subcategory** | **Diabetes mellitus arising in pregnancy** | **JA63.2** | **1320503631** |
| **Subcategory** | **Other specified diabetes mellitus in pregnancy** | **JA63.Y** | **/** |
| **Subcategory** | **Diabetes mellitus in pregnancy, unspecified** | **JA63.Z** | **/** |
| **Subcategory** | **Neonatal diabetes mellitus** | **KB60.2** | **1217915084** |
| **Subclassification** | **Transient neonatal diabetes mellitus** | **KB60.20** | **1596856936** |
| **Subclassification** | **Other specified neonatal diabetes mellitus** | **KB60.2Y** | **/** |
| Foundation entities | Permanent neonatal diabetes mellitus | KB60.2Y | 33655955 |
|  | Syndromic permanent neonatal diabetes mellitus | KB60.2Y | 1271599739 |
|  | DEND - [Developmental delay - epilepsy - neonatal diabetes] syndrome | KB60.2Y | 31390672 |
|  | Permanent neonatal diabetes mellitus - pancreatic and cerebellar agenesis | KB60.2Y | 1871318195 |
| **Subclassification** | **Neonatal diabetes mellitus, unspecified** | KB60.2Z | **/** |
| **Subcategory** | **Wolfram syndrome** | **[5A61.5](https://icd.who.int/dev11/l-m/en#http://id.who.int/icd/entity/1009553897)** | **151381747** |
| **Subcategory** | **Maternally inherited diabetes and deafness** | **LD2H.Y** | **2133824111** |
| **Subcategory** | **Thiamine-responsive megaloblastic anaemia syndrome** | **5C63.Y** | **1165037235** |
| **Subcategory** | **Woodhouse-Sakati syndrome** | **5A61.0** | **1893572805** |
| **Subcategory** | **Mitochondrial myopathy with diabetes mellitus** | **8C73.Y** | **1930317843** |

Note: This table does not include diabetes-related chronic complications and prediabetes. Entities presented in bold constitute codable items for mortality and morbidity statistics per the ICD-11 2025 frozen version. Non-bolded terms represent Foundation entities that do not possess an independent, reportable MMS code. Instead, they are assigned a category code (e.g., all MODY subtypes fall under 5A13.6) and are precisely identified through their unique URIs, ensuring accurate documentation and interoperability.
